# Practical implementation of AI in a non‐academic, non‐commercial Pathology laboratory: Real world experience and lessons learned

**DOI:** 10.1111/his.15481

**Published:** 2025-06-17

**Authors:** Frederik Deman, Glenn Broeckx, Sabine Declercq, Quentin Degotte, Jakob Raymaekers, Roberto Salgado, Amélie Dendooven

**Affiliations:** ^1^ PA^2^, Department of Pathology Ziekenhuis aan de Stroom (ZAS) Antwerp Belgium; ^2^ Department of Diagnostic Sciences, Faculty of Medicine and Health Sciences University of Ghent Ghent Belgium; ^3^ Centre for Oncological Research (CORE), MIPPRO, Faculty of Medicine Antwerp University Antwerp Belgium; ^4^ Department of Mathematics University of Antwerp Antwerp Belgium; ^5^ Division of Research Peter Mac Callum Cancer Centre Melbourne Australia; ^6^ Department of Pathology University Hospital Ghent Ghent Belgium

**Keywords:** artificial intelligence, real world experience, workflow

## Abstract

**Aims:**

As pathology departments transition towards digital workflows, the integration of artificial intelligence (AI) is anticipated to become increasingly common. This study aimed to describe the real‐world implementation and impact of AI integration in routine pathological diagnostics, specifically focusing on prostate biopsy evaluations at the Department of Pathology, ZAS Hospitals, Antwerp.

**Methods and Results:**

An AI tool for analysing prostate biopsies was integrated into the department's daily workflow by embedding it into existing laboratory information and reporting systems. Following a short adaptation period, the use of AI led to measurable improvements. Most notably, there was a reduction in the number of immunohistochemical tests required, indicating more confident primary diagnoses. Additionally, a significant decrease in turnaround times for biopsy evaluations was observed, highlighting improved efficiency. The implementation process was closely monitored, and practical insights were gathered to guide future AI deployments in pathology.

**Conclusions:**

The first‐year experience of integrating AI into daily pathological practice demonstrated tangible benefits in diagnostic efficiency and workflow optimization. However, the process also revealed several challenges related to real‐world deployment, including adaptation by staff and system integration hurdles. The lessons learned provide valuable guidance for other institutions considering similar AI implementations, reinforcing the importance of strategic planning, training and system compatibility in successful adoption.

## Graphical abstract

Technical flow of the AI integration. Whole Slide Image (WSI); Image Management System (IMS); Laboratory Information System (LIS); Electronic Patient Record (EPR).

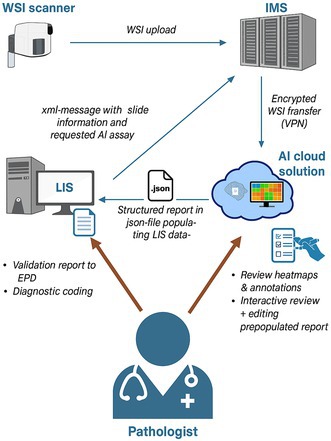

**Abbreviations**
AI: artificial intelligence
API: application programming interface
CE‐IVD: Conformité Européenne – In Vitro Diagnostic
CPIO: Chief Pathologist Information Officer
DPIA: data protection impact assessment
EHR: Electronic Health Record
GDPR: General Data Protection Regulation
HE: haematoxylin and eosin
ICT: Information and Communication Technology
IHC: immunohistochemistry
IMS: Image Management System
IP: Internet Protocol
LIS: Laboratory Information System
MSI: Microsatellite Instability
TAT: turnaround time
TIA: transfer impact assessment
VPN: virtual private network
WSI: whole slide image

## Introduction

As pathology departments increasingly transition from traditional microscopy to digital workflows, AI technologies have the potential to increase efficiency and diagnostic accuracy.^1^, ^2^, ^3^, ^4^, ^5^

Reports detailing the practical implementation of AI in the everyday workflow of pathologists are, to the best of our knowledge, non‐existent. This lack of documentation hinders their broad adoption.

AI provides a range of sophisticated tools that may enhance and support image‐based decision‐making.^3^, ^4^, ^5^, ^6^ Computational models analyse vast amounts of digital pathology images with remarkable speed and precision, identifying subtle patterns and anomalies that might escape the human eye. This capability not only accelerates the diagnostic speed, it also provides pathologists with valuable secondary (AI‐)opinions, reduces diagnostic errors leading to improved patient outcomes.^7^, ^8^ Moreover, AI‐driven image analysis can quantify cellular features, tissue structures, and biomarkers with a level of consistency and objectivity that surpasses that of manual assessment.^9^, ^10^ Additionally, AI systems can be employed for slide quality control purposes such as continuous monitoring of diagnostic results to ensure accuracy and consistency across multiple cases and pathologists.^11^, ^12^, ^13^

AI systems can optimize workflow management, prioritize urgent cases, and allocate resources efficiently. By automating certain tasks and improving efficiency, AI systems can alleviate the workload of existing pathologists and increase the capacity of pathology laboratories.^14^, ^15^

However, the integration of AI into pathology laboratories comes with obstacles. These include the requirement for costly and advanced digital imaging equipment, such as whole‐slide imaging scanners, and a reliable data storage system capable of handling large quantities of digital pathology images. Additionally, there is a need for compatibility between various software systems, including Laboratory Information Systems (LIS) and Image Management Software (IMS).^16^ Ethical considerations must also be addressed, such as implementing measures to safeguard patient privacy and ensuring secure management of sensitive medical information.^17^ Furthermore, it is essential to establish clear protocols governing the use of AI in clinical decision‐making to ensure that AI‐generated results are presented transparently, minimizing the risk of cognitive biases in diagnosis. These protocols should emphasize explainability, validation against established medical standards, and clinician oversight to prevent over‐reliance on AI.

In 2021, a large hospital network in the Antwerp region was created. The pathology laboratory PA^2^ (Ziekenhuis aan de Stroom (ZAS), Antwerp) was in charge of delivering pathology services to the participating hospitals, across multiple sites. This was the driver for digitalizing our services. Subsequently, three Leica Aperio scanners (Leica Biosystems, Wetzlar, Germany) were purchased with server space for 3‐month storage of whole‐slide images. Venturing AI solutions was the logical next step.

We first implemented in 2023 the Galen™ Prostate‐assay, in the laboratory's daily workflow.^18^ Currently, different AI tools such as Galen™ Prostate, Galen™ Breast, Galen™ Gastric, Mindpeak PDL1, Aiosyn mitotic figure counting, Owkin BreastRlaps, PRIMAA Cleo skin, and Hologic Genius have been explored.

In order to facilitate exploring partnerships with industry, a Study and Innovation Unit dedicated to partnerships with commercial partners such as AstraZeneca (UK), Johnson and Johnson (US), Datexim (France), Mindpeak (Germany), Owkin (France), PRIMAA (France) and IBEX (Israel) was installed.

While numerous laboratories have published on the digitization of their workflows, reports detailing the practical implementation of AI in the everyday workflow of pathologists are, to the best of our knowledge, non‐existent.^19^, ^20^, ^21^ This manuscript aims to address this gap by sharing a real‐world experience. We restrict “AI” in our paper to image‐analysis algorithms that assist pathologists during the primary diagnostic evaluation of whole‐slide images. Applications such as case‐prioritization before first assessment, speech‐recognition for report generation, or automated quality‐assurance dashboards–although also AI‐enabled–are outside the scope of this manuscript.

## Methods: Implementation Process

### Market Research and Selection of Tools of Interest

In 2021, we conducted market research involving 15 AI companies, focusing on commercially available AI tools. The companies and their tools were evaluated using test batches of 20 different slides for different use cases of relevance to the daily practice. The test batches include samples with different levels of complexity, such as cases that present diagnostic difficulties, like those with rare diagnoses or slides containing a minimal amount of tumoral tissue. Each company's maturity was evaluated using the following variables: (1) The time since start‐up of the company, (2) The number of assays available, (3) The presence of assay certification by regulatory bodies, (4) The experience installing AI tools in a daily workflow, (5) The presence of a strategy on interoperability with software packages, such as the LIS or IMS.

A shortlist of companies for different use cases was subsequently created. There was no commercial or financial interest driving the selection of these partnerships. Criteria‐driven evaluations were integrated in the agreements with all companies.

### IT Infrastructure and IT Security

**Figure 1:**
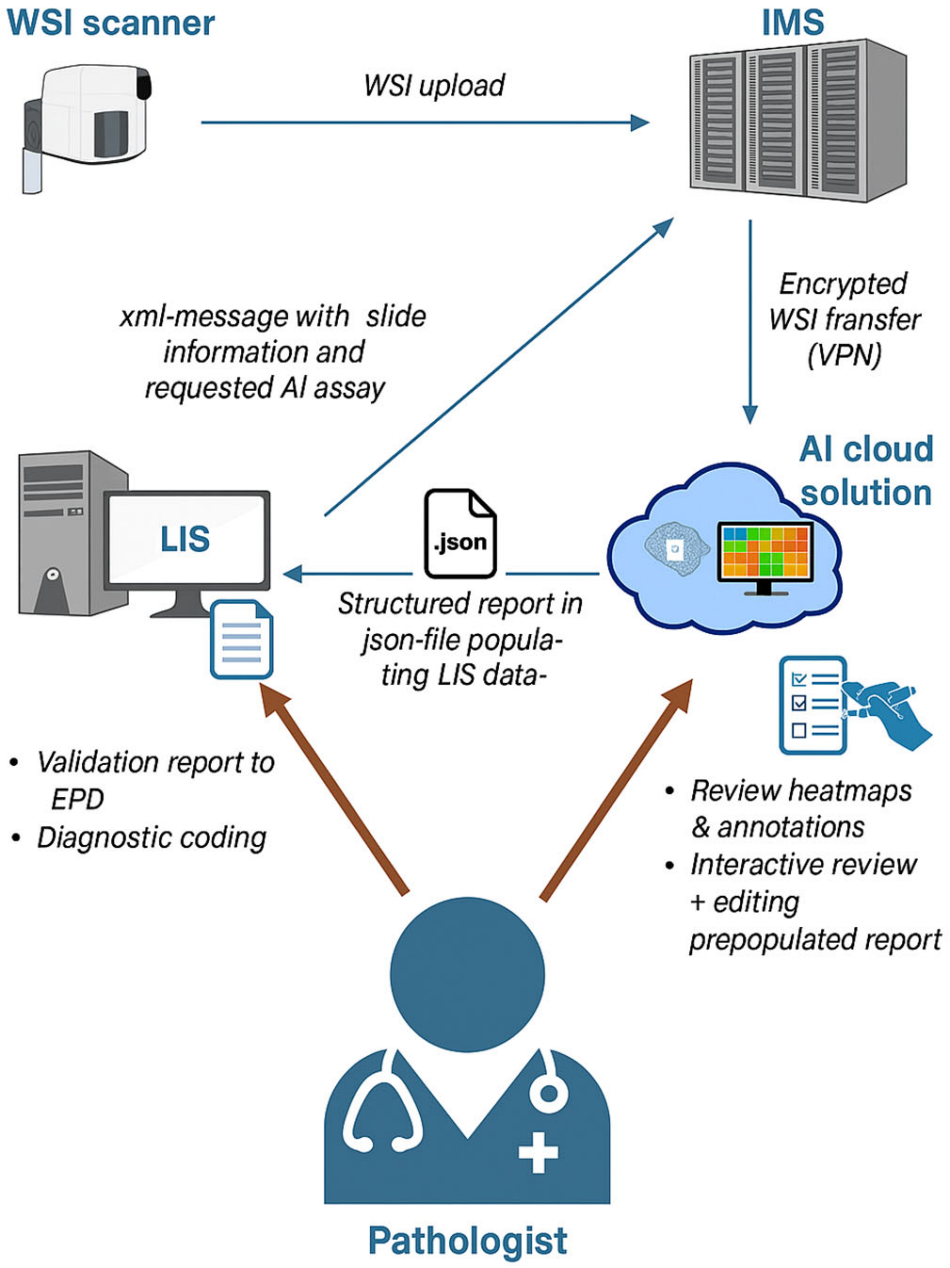
Technical flow of the AI integration. IMS, image management system; LIS, lab information system; POI, points of interest; WSI, whole slide image.

A summary of the whole technical implementation is shown in Figure 1.

Before implementation, an IT infrastructure and security framework is needed. Two primary deployment models can be considered: (1) cloud‐based and (2) on‐premises installations, the latter meaning an installation on servers in a physical lab or hospital environment. The relevant differences between the two options are shown in Table S1. The choice between cloud and on‐premises AI solutions should factor in operational needs, budget constraints, regulatory requirements, and the lab's long‐term strategic goals. Some AI vendors offer a choice between cloud installation and on‐premises installation; however, most vendors offer only a cloud‐based solution. In our experience, on‐premises installation comes with high extra costs. This can create a bottleneck in the workflow when multiple AI analyses are used simultaneously.

To connect to the cloud, the following three methods have been proposed by one or more AI vendors, with each one requiring specific security measures. These were approved by the ICT data security officer of the hospital. The following list, in order of preferred solutions, provides a summary of possible cloud connections:

1. VPN tunnel: this is a secure, encrypted connection between a dedicated server in the internal network—in our specific IT configuration named ‘AI‐flow‐server’—and a remote server that enables secure data transmission over the Internet. A group of security protocols is used to establish and run this VPN connection where this group ensures authentication data encryption and security association by running on top of the Internet Protocol (IP). It creates a virtual private network that encapsulates data packets, thereby ensuring confidentiality and integrity. The use of this ‘AI‐flow’ server is discussed further in the section about the IMS integration.
2. IP whitelisting over the public Internet: this system restricts access to systems based on approved IP addresses, thereby enhancing security. Additional measures to secure data transfer are necessary for comprehensive protection.
3. Specific software of the AI vendor: AI vendors often require on‐premises software to connect with their cloud environment, necessitating an extra dedicated server and increasing implementation costs. This solution requires additional security measures to secure the data transfer.

### IMS Integration

Digital images are viewed and managed through an IMS; we use Telemis, Belgium. It is highly desirable to achieve seamless integration of AI systems within the IMS to enhance workflow efficiency. This leads to a better user experience. We installed a separate server (‘AI‐flow‐server’), to manage the flow of whole‐slide images (WSI) to the different AI tools. This guarantees the integrity of the routine slide assessment workflows and the use of the IMS‐viewer, being separate from the AI‐workflow. The segregation of AI processing manages computational resources more effectively, preventing slowdowns in the primary IMS viewer.

The tight integration between IMS and AI systems, with the integration of graphic AI elements in the primary IMS viewer, raised concerns about the CE‐IVD label for each assay, for example, when the AI vendor provides an associated viewer (as part of the global CE‐IVD label of the assay) different from the local IMS viewer. Conceptually, regulatory bodies may require additional validation and certification processes after the integration of an AI tool within the IMS viewer to ensure that the integrated AI components do not compromise the diagnostic accuracy or reliability of the system. Embedding the AI viewer in the primary IMS environment may be a solution to this conundrum.

In our context, the IMS supports the LIS to ensure a LIS‐driven digital pathology workflow (Figure 1). This ensures that the LIS functions as the digital center of the lab, directing and supporting various laboratory workflows. When integrating AI tools into the workflow, the IMS ensures that the workflow runs optimally by sending commands to the different AI‐assays. Therefore, additional links between the LIS and IMS are necessary for exchanging XML messages directing the IMS. Specifically, the LIS‐driven link to the IMS viewer generates case‐specific attributes with information on whether the slides should be sent for AI analysis or not, to which vendor the slides should be sent, and which AI‐assay should be applied. Distributing slides to different AI‐assays required additional IT development in the IMS (thus costs) to ensure the monitoring of access to images and the continuous validation of the retrieval and sending of images to specific AI tools, allowing full traceability of slide requests and access by external parties.

### LIS Integration

The IBEX AI‐assays offer a structured reporting functionality in the viewer, which was nearly identical to the one we used in our LIS, facilitating integration of the two systems. A web service was built based on the application programming interface (API) provided by the AI vendor. This API directly and automatically integrates AI results, including corrections made by the pathologist, into the report in the LIS and into the underlying database after validation in the AI cloud environment.

**Figure 2:**
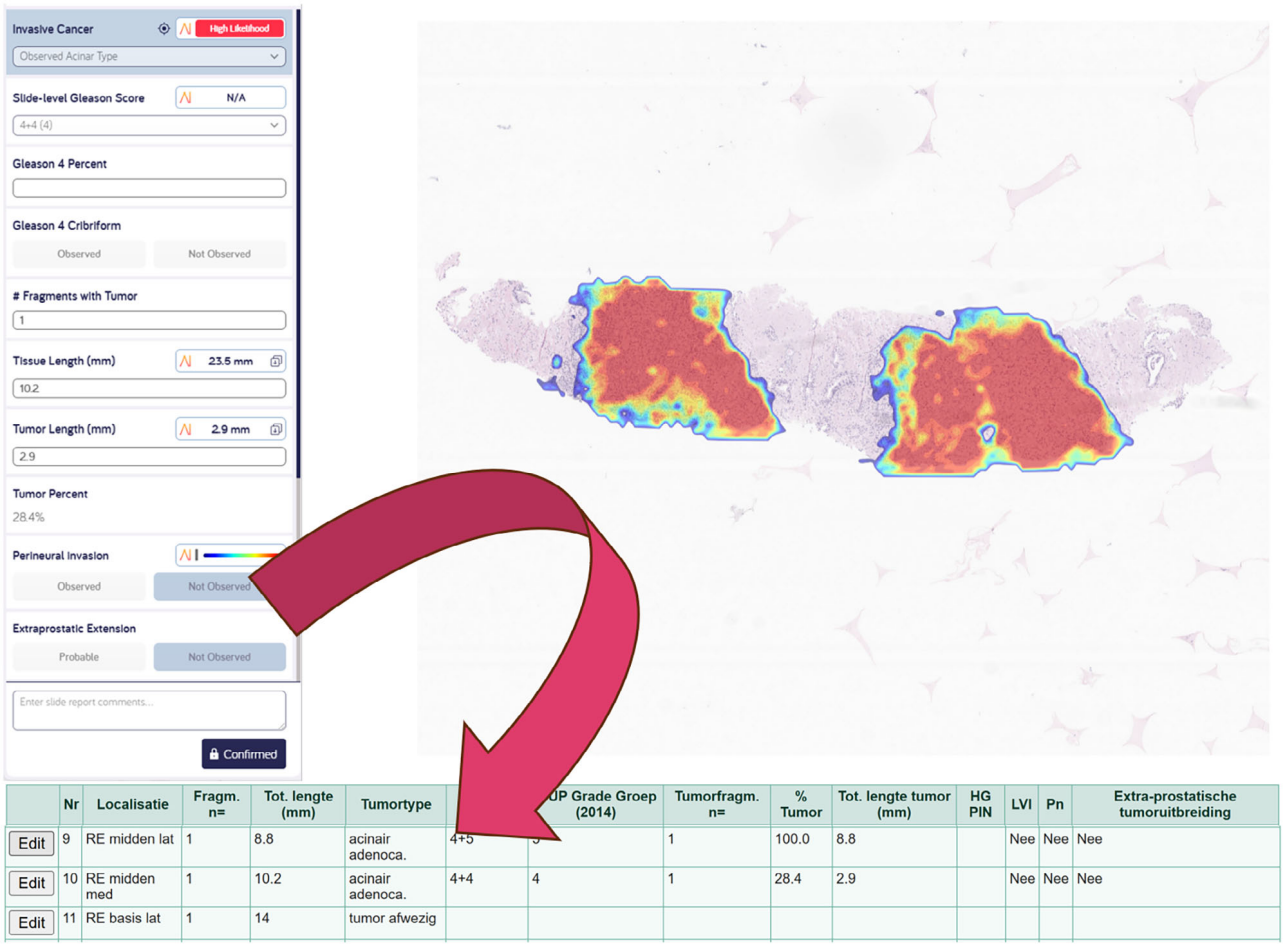
Schematic overview of the AI–LIS integration.

Evaluation of the AI‐generated suggestions through heat maps within the cloud‐based AI platform (and the partially pre‐populated structured report) assists the reviewing pathologist in the diagnostic assessment. Following review and potential adjustments, the pathologist validates the report. This triggers the transmission of structured data (e.g. including findings, categorizations, and pathologist comments) to the LIS. This data is then used to populate the report and the underlying database in the LIS, enabling the report's transmission to the Electronic Health Record (EHR) which is demonstrated in Figure 2. This transmission is done by the exchange of a case‐specific JSON file from the AI tool with the LIS. The technical flow with digital messages between the AI tool and the LIS is shown in Figure 1. This integrated setup helps to reduce the administrative workload associated with pathologists' reporting duties.

The exchange of data between the AI cloud environment and the LIS sets the stage for future AI applications. These may include worklist management, automatic requesting of additional techniques based on a certain probability of the presence or absence of lesion types or malignancy, or triage for additional biomarker determination, such as MSI testing in colorectal carcinomas based on an AI assessment of haematoxylin and eosin staining (HE).

### Data Security and GDPR

The choice of preferred cloud solutions needed documentation in order to comply with GDPR regulations. More specifically, a data protection impact assessment (DPIA) and a transfer impact assessment (TIA) was required. The data security policy was established in close collaboration with the Data Protection Officer of ZAS.

### Data Protection Impact Assessment (DPIA)

Data Protection Impact Assessment (DPIA) is a process designed to help organizations identify and mitigate the data protection risks associated with their activities, particularly in accordance with the General Data Protection Regulation (GDPR). The following topics were handled in the DPIA:

1. The goal of the project is to implement AI tools in the clinical routine of the pathology lab with evaluation of the potential impact on the care process of patients. A quadruple aim scheme^22^ was used to answer this question (see Table S2).
2. A systematic description of the data processing pipeline included a description of the persons of interest involved, the technology used, the processing steps, and a chart with data flows.
3. The necessity and description of alternatives, including questions such as the consent of persons whose data will be processed, the information process of the involved parties, a description of the quality of the data used during the project, how long data are stored, how data confidentiality is guaranteed, whether specific security measures are taken, and the possibility of reusing data for secondary purposes.
4. Justification of need and alternatives: again, we used the quadruple aim scheme to justify the use of AI in clinical practice, including expected health improvement by better accuracy, a decreased TAT, possible healthcare cost efficiency by automation of lab processes, and improvement in the experience of healthcare providers by a decreased working pressure.
5. The possible negative consequences of using AI for individuals include data leakage by the cloud provider and the hazard of incorrect information provided to the pathologist by the AI tool. A risk assessment was used to address these issues. In addition, a roundtable conference was organized to obtain patient representatives' opinions on the use of AI and its possible negative consequences.
6. Measures considered or taken to address risks include the removal of patient names from slide labels, ICT‐security measures (taken by both the AI vendors and the ICT management of the laboratory), increasing transparency to the patient by explaining the usage of AI on the website of the lab, including the usage of AI in the general terms of the hospital, and technical validation procedures before implementation of the AI tools in routine clinical practice.
7. The opinion of stakeholders about the risks: for this, we organized a roundtable conference with patient representatives from different oncology entities. Here, patient representatives indicated that they preferred increased accuracy and speed of diagnosis by using innovative technology over risks related to data security and privacy. According to patients, TAT is one of the key quality indicators for laboratories.
8. Advice from the DPO regarding the DPIA. This advice further considers whether the DPIA is in line with Article 35 of the European GDPR. A review was recommended every 2 years. A new evaluation of the DPIA will be performed for each new AI tool that will be implemented in clinical routine.
9. As an attachment, we include extra documentation of the IT security of AI vendors.

### Transfer Impact Assessment (TIA)

The Court of Justice of the European Union (C‐311/18, Schrems II) reaffirmed that personal data in the European Economic Area (EEA) must be protected, regardless of location. Exporters of data are responsible for assessing whether third‐country laws or practices affect the effectiveness of the GDPR‐approved safeguards on a case‐by‐case basis. If so, exporters can implement additional measures to ensure compliance with EU security standards. A TIA is a questionnaire that helps evaluate the risk related to the transfer of personal data and whether this risk can be accepted.

### Training

Despite rapid advancements in AI technology and its increasing adoption in various healthcare settings, the pathology community generally lacks computational expertise. At the moment of implementation, there were no specific, comprehensive frameworks to train pathologists, technicians, and support staff in operationalizing AI tools. This gap not only hampers the optimization of AI systems but also raises concerns regarding the reliability and interpretability of AI‐generated insights, as inadequate training may lead to misuse, misinterpretation, and variability in clinical outcomes.^23^, ^24^

We initiated a comprehensive training program for staff that focused on the application of artificial intelligence (AI) in the field of pathology. This program began with a general information session that outlined various AI technology use cases. Subsequently, pathologists were offered more in‐depth training sessions, developed in close collaboration with the commercial vendors responsible for implementing AI tools. To develop training content, we drew upon other guidelines established in other related disciplines.^25^, ^26^ The training content is shown in Table S3. Pathologists must understand the implications of statistical metrics linked to the AI tool. For example, in cases in which an algorithm is designed primarily for high sensitivity, this high sensitivity may lead to an increased incidence of false‐positive results, and it is thus important that pathologists are aware of this risk when they apply an AI‐assay.

The thorough integration of AI tools within our laboratory information system (LIS) and the accompanying reporting framework facilitated the collection of feedback on the performance of tools relative to the pathologists' findings. During the evaluation, pathologists were presented with their own reports alongside the AI assessments of cancer likelihood, categorized as high, medium, or low. This feedback was deemed valuable by pathologists, enhancing their confidence in understanding the functionality of the AI tool and establishing its accurate and safe use.

### Validation

The validation process for the clinical application of AI tools faces significant challenges owing to the absence of established guidelines. Our validation protocol comprised a technical assessment conducted in collaboration with a commercial AI vendor (IBEX), which included the assembly of an in‐house collected retrospective dataset of 20 cases reflecting a variety of diagnoses. The results generated by the AI tool were systematically compared with the historical diagnoses and evaluations by external pathologists affiliated with the AI vendor, achieving a concordance rate of 100%. This number was selected after a prospective risk analysis that balanced potential diagnostic impact, morphologic diversity and routine workload. It also aligns with the Leeds Digital Pathology Group's Practical Guide to the Implementation of Digital Pathology for Primary Diagnosis, which recommends a 20‐case training‐validation set.^27^ Importantly, the AI provides only supplementary visual cues (heat maps and region annotations) superimposed on the whole‐slide image. The underlying, unaltered morphology remains fully accessible, so any residual risk from algorithmic error is limited and the pathologist retains final authority. In effect, every slide is assessed both with and without the AI overlay, creating a continuous, case‐by‐case validation rather than a one‐off exercise tied to a fixed number of cases, as suggested in guidelines for a digital‐pathology roll‐out.^27^, ^28^ Furthermore, the validation documentation included a comprehensive risk analysis that emphasized the primary role of the pathologist, positioning the AI tool as a decision support system rather than a replacement. The incorporation of visual feedback on the algorithm performance was designed to mitigate potential risks. However, it is essential to note that any AI tool that assumes an active decision‐making role within the patient‐care continuum requires more stringent validation protocols.

## Methods: Proof‐of‐Concept Study

To evaluate the comprehensive effects of integrating artificial intelligence (AI) into clinical practice, we conducted an observational retrospective study complemented by a qualitative assessment of user interactions using an AI tool designed for the evaluation of prostate needle biopsies. This retrospective analysis focused on two primary outcome measures: turnaround time (TAT), defined as the period from the assignment of a case to a pathologist following scanning until the initial validation report, and the incidence of additional diagnostic procedures utilized during the work‐up. The study delineated a 3‐month implementation and adaptation phase, followed by a subsequent evaluation period. We conducted an independent *t*‐test using SPSS to compare the two analogous time frames: the first reflected a complete digital workflow prior to the implementation of the AI tool for prostate biopsies, whereas the second analysed the same workflow following its implementation. The evaluation periods ran from January 2023 to December 2023 (*n* = 528) in comparison with January 2024 to December 2024 (*n* = 526).

## Results

Following the implementation of AI technologies, we observed a significant increase of approximately 7 h in TAT during the initial adaptation phase in 2024 (*n* = 155) compared to 2023 (*n* = 170). This was anticipated due to the learning curve associated with new technology adoption. In contrast, TAT exhibited a significant decline later in 2024 (*n* = 371) compared to the same time frame in 2023 (*n* = 358), with an average reduction of approximately 9 hours, equivalent to a gain of approximately one working day. Additionally, both during the adaptation phase and subsequently, there was a significant reduction in the number of supplementary immunohistochemical tests required for the diagnostic evaluation of prostate biopsies compared with 2023 and 2024, with a decrease of approximately one‐third in test utilization in both the adaptation and follow‐up periods. Results are demonstrated in Table 1. Detailed results per quarter are shown in Tables S4 and S5.

**Table 1:** Mean number of Immunohistochemical tests (IHC) and Turn Around Time (TAT) for each prostate biopsy specimen during the initial adaptation period (Q1 2024; *n* = 155) and subsequent period (Q2–Q4 2024; *n* = 371) following the introduction of an artificial intelligence system for prostate biopsy evaluation, contrasted with the corresponding timeframe in the previous year (Q1 2023; *n* = 170 and Q2‐Q4 2023; *n* = 358).

	Reference period (2023)	AI in clinical routine (2024)	*p*‐value
**Number of IHC slides per case Adaptation period Q1**	2.98 ± 0.16	2.26 ± 0.18 ↓	0.001
**Number of IHC slides per case Q2–Q4**	2.89 ± 0.11	2.13 ± 0.11 ↓	<0.001
**TAT adaptation period Q1 (days)**	1.65 ± 0.08	1.94 ± 0.10 ↑	0.014
**TAT Q2–Q4 (days)**	2.09 ± 0.06	1.71 ± 0.06 ↓	<0.001

## Discussion

The integration of artificial intelligence (AI) in pathology holds great promise for improving diagnostic accuracy, reducing workloads, and enhancing overall efficiency. However, its practical implementation presents several challenges that must be addressed before adoption.^4^, ^29^, ^30^ To help the implementation of AI tools in other labs, we made a summary of the lessons learned in Table 2, and propose solutions.

**Table 2:** Resume of difficulties encountered during implementation of AI in the clinical routine of a lab together with possible solutions.

**ICT architecture**	Connection to third party	Installation and documentation of VPN tunnel Documentation of additional measures for IP‐whitelisting Documentation of additional measures for specific vendor software
	Lean ICT flow for AI	Installation of extra AI‐server apart from the IMS
**Software development** **–** **LIS**	Trigger points to send slides to AI server	Specific stain All HE slides of a case, based on report/sample type Specific slide of a case based on trigger in macroscopy Manual trigger
		Standardization of communication between LIS and IMS by .xml messages
	Deep integration of AI results	Alignment of report content
		Standardization of communication between AI and LIS by .json messages
**Software development IMS**	Instal WSI flow to AI service	Configuration .xml message of LIS and appropriate actions to give AI tool access to a specific WSI
	Validation access to images	Create register of accessed slides by third party with validating if appropriate LIS‐order exists.
	Integrate AI results in viewer	Document standardized data format for POI or heatmaps
**GDPR administration**	Patient opinion about implementation of diagnostic AI tools	Organization of round table conference about AI and digital pathology
	Define patient identifiers in the dataflow	Expert opinion statement about lab‐identifiers and information in WSI that can identify individual persons Remove patient names from slide labels
**Patient information**	Inform patients on the use of AI in clinical routine	Publish statement on the lab website for patients Put additional information in the hospital general terms for patients
**Validation**	Lack of concrete guidelines	Risk assessment on validation process and clinical use as a decision support tool
**Training**	Lack of concrete guidelines	Rely on AI vendor experience Look into guidelines from other medical disciplines Creation of feedback loops for continuous training and support

Abbreviations: IMS, image management system; LIS, lab information system; POI, points of interest; WSI, whole slide image.

One of the critical hurdles is the lack of standardization of AI results, in particular the interoperability of different systems.^4^ Without standardization, AI tools risk generating results that cannot be easily interpreted across platforms or require manual adjustments, undermining efficiency. Standardization of AI results is crucial to enable interaction with other software packages used in pathology laboratories, optimizing the overall workflow. Although digital pathology is often perceived as time‐consuming, the integration of AI can mitigate this delay, as we show in this paper. By automating routine tasks and providing pathologists with accurate and timely information, AI can potentially make digital workflows more efficient than traditional analog processes, leading to quicker diagnoses and improved clinical outcomes. Thus, standardizing AI results is not just a technical necessity but a key factor in realizing its full potential.

Another significant challenge with implementing commercially available AI tools in pathology laboratories is the reliance on cloud‐based solutions, which introduce complex issues related to data governance and regulatory compliance. A primary concern is ensuring AI solutions comply with stringent regulations such as the General Data Protection Regulation (GDPR). Most pathology laboratories lack the expertise and resources to effectively manage these compliance processes, creating a substantial burden. The workload is amplified when multiple AI tools from different vendors are implemented, as each requires its own compliance procedures, leading to repetitive tasks for lab staff. This lack of familiarity with regulatory requirements, combined with the time and resources needed for navigation, can significantly hinder AI adoption in clinical practice. A potential solution to streamline this process could be introducing AI brokers and middleware platforms as intermediaries between laboratories and AI vendors. These brokers would manage administrative processes related to privacy and ICT security, ensuring all AI integrations adhere to relevant regulations. By centralizing compliance tasks with a single point of contact, laboratories would only need to engage in the administrative process once, reducing the burden on staff. This approach can enhance the efficiency of AI adoption while ensuring privacy and security standards are consistently met. Alternatively, we support the introduction of a Chief Pathologist Information Officer (CPIO) as suggested by RCPath Australasia in their AI guidelines. The CPIO role is emerging as critical to guide the integration and utilization of artificial intelligence in pathology laboratories. This leadership role bridges pathology expertise and information technology, overseeing the implementation of AI tools and digital pathology systems. The CPIO is responsible for evaluating AI algorithms, ensuring their clinical validity and utility, and developing protocols for their integration into existing workflows.^31^

The ambiguity surrounding cloud‐based storage solutions for clinical AI is striking. Cloud storage offers the advantage of lumping technical resources for fast image analysis, but introduces inefficiencies when combined with local storage systems used for clinical routine. Often, digital pathology images are temporarily stored in the cloud for AI processing, separate from the storage used for Whole Slide Images by an IMS. This creates a situation where data are duplicated and stored both in the cloud and locally, raising concerns about data management, security, and cost. The storage of large digital images required for pathology can be extremely expensive, due to their size.^30^

During the implementing of artificial intelligence (AI) we faced the lack of established training and validation guidelines. The Royal College of Pathologists of Australasia has addressed this by developing guidelines incorporating a risk‐based approach for validation and quality assurance, with practical implementation considerations.^31^ Other professional societies need to provide practical guidance on training, deployment, and quality assurance for integrating AI into clinical pathology practice. The College of American Pathologists (CAP) has introduced guidelines for evaluating AI algorithms, but these focus primarily on technical and statistical performance evaluation, with limited practical guidance on training and quality assurance.^32^ This makes them less applicable for laboratories lacking specialized AI knowledge or experience implementing innovative techniques. Developing comprehensive guidelines is crucial, as they must account for specific algorithm use cases and associated risks. For example, an AI tool making autonomous decisions without visual feedback presents different challenges compared to those using heatmaps or points of interest on WSIs. Without a robust framework addressing these nuances, AI integration into pathology workflows may be hindered, potentially compromising diagnostic reliability and safety. Therefore, developing tailored guidelines focusing on both technical aspects and clinical implications is essential.

We have done a preliminary real‐world validation in prostate cancer in the daily workflow. Previous workflow studies conducted in controlled environments indicated a decrease in hands‐on time.^33^, ^34^ A recent prospective trial was unable to verify this reduction in hands‐on time.^15^ A reduction in techniques required was notable, observed early during the adaptation period. This is significant given that laboratory immunohistochemistry techniques are reimbursed by healthcare insurers but not AI, making our findings impactful from clinical and financial standpoints. Our findings confirm reports from other authors of similar reductions in immunohistochemical tests.^15^, ^33^, ^34^, ^35^, ^36^ Notably, the variability observed between the 3‐month adaptation phase and the remainder of the year is of similar magnitude to the variability recorded between intervals with and without AI support. A plausible explanation is the markedly greater on‐site presence of uropathologists during Q1 2023 relative to the rest of the study period due to personal planning in the laboratory. This increased staffing level likely confounds— and therefore tempers— the apparent rise in turnaround time (TAT) seen when comparing the pre‐AI and post‐AI introduction. The reduction in additional techniques requested, while maintaining diagnostic confidence using AI, could be the primary reason for a shorter turnaround time. However, this decrease in TAT was not immediate. The increase in TAT during the initial adaptation period can be explained by the learning curve associated with implementing a new system. Pathologists need time to become familiar with the tool, build confidence in its output, and integrate it into their workflow. Over time, as proficiency increased, efficiency gains became evident, validating the long‐term potential of AI. This preliminary real‐world analysis suggests that AI use in daily practice can optimize workflow and reduce costs. These findings need confirmation in prospective studies, which are currently scarce in literature.^37^

A critical factor for the broader implementation of AI in the pathology community is the need for real‐world data.^30^ Technologies' true effectiveness can only be fully understood when integrated into routine practice. To facilitate widespread AI adoption in pathology, it's essential to create a collaborative environment where laboratories share real‐world experiences with the broader community. Insights from practical applications can offer valuable lessons on AI tool performance in diverse settings under varying conditions and workflows. Understanding challenges faced by laboratories can help develop strategies to overcome obstacles and improve technology. Sharing successful use cases can provide a roadmap for fellow institutions, accelerating adoption. By fostering open exchange of experiences, pathology laboratories can collectively address barriers to AI integration, ensuring tools are safe, effective, and optimized for widespread use across diverse clinical environments.

Pathologists should engage with AI in pathology by embracing AI to enhance diagnostics and efficiency, adapting to digital workflows and AI analyses, and critically evaluating AI output.^38^ They should advocate for proper infrastructure, develop ethical guidelines for AI use, and contribute to AI validation and quality control. Additionally, pathologists should focus on complex case interpretation and adapt their roles to leverage AI effectively, ensuring human expertise remains central to diagnostics while benefiting from technological advancements.

### Perspectives

The implementation of artificial intelligence (AI) in pathology laboratories represents a significant advancement in diagnostic medicine, offering potentially improved accuracy, efficiency, and workflow optimization. However, the integration of AI tools into clinical practice presents several challenges that require careful consideration and strategic solutions. Key issues include the need for standardization of AI outputs, data governance and regulatory compliance concerns with cloud‐based solutions, storage ambiguities, and a lack of established guidelines for training and validation. Despite these challenges, a preliminary analysis of the integration of an AI tool for prostate biopsies in our lab has demonstrated promising results, including reduced turnaround times and fewer stains needed. To fully realize the potential of AI in pathology, standardized protocols are needed, addressing data security concerns as well as training and validation. In addition, sharing real‐world experiences among pathology laboratories will be instrumental in overcoming obstacles and accelerating the adoption of AI technologies. As the field continues to evolve, ongoing collaboration between pathologists, AI developers, and regulatory bodies will be essential to ensure the responsible and effective integration of AI into pathology practice. This collaborative approach will enhance diagnostic capabilities and contribute to improved patient care in the rapidly advancing landscape of digital pathology.

## Conclusion

Implementing an AI‐assisted prostate‐biopsy workflow in a medium‐volume, non‐academic pathology laboratory proved both technically feasible and clinically beneficial. While initially presenting a learning curve evidenced by a temporary increase in TAT, it ultimately resulted in a significant reduction in TAT by approximately 9 h and a decrease in ancillary immunohistochemical testing by roughly one‐third after an adaptation period. Our experience in the implementation process underscores the importance of addressing IT infrastructure, LIS integration, data security, and staff training for successful AI adoption. The framework and lessons learned offer a practical template for other laboratories considering similar AI implementations and underscore the need for broader prospective studies to confirm effectiveness and generalizability across use cases.

## Author contributions

Conceptionalizing: FD, AD. Data collections: FD. Statistics: FD, GB, QD, JR. Writing: FD, GB, AD. Proofreading: FD, GB, AD, QD, SD, RS, JR.

## Funding information

This study was not supported by any funding.

## Conflict of interest statement

The authors declare no relevant conflicts of interest related to this study.

## Patient consent statement

As this is a retrospective study using anonymized data, the requirement for informed consent was waived.

## Supporting information


Data S1.

## Data Availability

All data generated or analysed during this study are included in this published article and its supplementary information files or are available from the corresponding author upon reasonable request.

## References

[1] Wong ANN , He Z , Leung KL *et al*. Current developments of artificial intelligence in digital Pathology and its future clinical applications in gastrointestinal cancers. Cancer 2022; 14; 3780. 10.3390/cancers14153780 PMC9367360 35954443

[2] Berbís MA , McClintock DS , Bychkov A *et al*. Computational pathology in 2030: A Delphi study forecasting the role of AI in pathology within the next decade. EBioMedicine 2023; 88; 104427. 36603288 10.1016/j.ebiom.2022.104427 PMC9823157

[3] Serag A , Ion‐Margineanu A , Qureshi H *et al*. Translational AI and deep learning in diagnostic Pathology. Front. Med. 2019; 6; 6. 10.3389/fmed.2019.00185 PMC6779702 31632973

[4] Cheng JY , Abel JT , Balis UGJ , McClintock DS , Pantanowitz L . Challenges in the development, deployment, and regulation of artificial intelligence in anatomic Pathology. Am. J. Pathol. 2021; 191; 1684–1692. 33245914 10.1016/j.ajpath.2020.10.018

[5] Current and future applications of artificial intelligence in pathology: A clinical perspective. J. Clin. Pathol. 2021; 74; 409–414. 32763920 10.1136/jclinpath-2020-206908

[6] Rodriguez JPM , Rodriguez R , Silva VWK *et al*. Artificial intelligence as a tool for diagnosis in digital pathology whole slide images: A systematic review. J Pathol Inform 2022; 13; 100138. 36268059 10.1016/j.jpi.2022.100138 PMC9577128

[7] Steiner DF , MacDonald R , Liu Y *et al*. Impact of deep learning assistance on the histopathologic review of lymph nodes for metastatic breast cancer. Am. J. Surg. Pathol. 2018; 42; 1636–1646. 30312179 10.1097/PAS.0000000000001151 PMC6257102

[8] Raciti P , Sue J , Retamero JA *et al*. Clinical validation of artificial intelligence–augmented Pathology diagnosis demonstrates significant gains in diagnostic accuracy in prostate cancer detection. Arch. Pathol. Lab Med. 2023; 147; 1178–1185. 36538386 10.5858/arpa.2022-0066-OA

[9] Abele N , Tiemann K , Krech T *et al*. Noninferiority of artificial intelligence–assisted analysis of Ki‐67 and estrogen/progesterone receptor in breast cancer routine diagnostics. Mod. Pathol. 2023; 36; 100033. 36931740 10.1016/j.modpat.2022.100033

[10] Bulten W , Balkenhol M , Belinga J‐JA *et al*. Artificial intelligence assistance significantly improves Gleason grading of prostate biopsies by pathologists. Mod. Pathol. 2021; 34; 660–671. 32759979 10.1038/s41379-020-0640-y PMC7897578

[11] Weng Z , Seper A , Pryalukhin A *et al*. GrandQC: A comprehensive solution to quality control problem in digital pathology. Nat. Commun. 2024; 15; 10685. 39681557 10.1038/s41467-024-54769-y PMC11649692

[12] Rodrigues D , Reinhard S , Waldburger T *et al*. Abstract 5442: SlideQC: An AI‐based tool for automated quality control of whole‐slide digital pathology images. Cancer Res. 2023; 83; 5442.

[13] Baba O . Qualitopix: Artificial intelligence‐based quantitative quality assurance of immunohistochemistry staining‐the Henry ford health experience. Am. J. Clin. Pathol. 2023; 160; S101–S102.

[14] Crowell EF , Bazin C , Saunier F *et al*. CytoProcessorTM: A new cervical cancer screening system for remote diagnosis. Acta Cytol. 2019; 63; 215–223. 30921788 10.1159/000497111

[15] Flach RN , van Dooijeweert C , Nguyen TQ *et al*. Prospective clinical implementation of Paige prostate detect artificial intelligence assistance in the detection of prostate cancer in prostate biopsies: CONFIDENT P trial implementation of artificial intelligence assistance in prostate cancer detection. JCO Clin Cancer Inform 2025; 9; e2400193. 40036728 10.1200/CCI-24-00193

[16] van Diest PJ , Flach RN , van Dooijeweert C *et al*. Pros and cons of artificial intelligence implementation in diagnostic pathology. Histopathology 2024; 84; 924–934. 38433288 10.1111/his.15153

[17] Chauhan C , Gullapalli RR . Ethics of AI in Pathology: Current paradigms and emerging issues. Am. J. Pathol. 2021; 191; 1673–1683. 34252382 10.1016/j.ajpath.2021.06.011 PMC8485059

[18] Pantanowitz L , Quiroga‐Garza GM , Bien L *et al*. An artificial intelligence algorithm for prostate cancer diagnosis in whole slide images of core needle biopsies: A blinded clinical validation and deployment study. Lancet Digit Health 2020; 2; e407–e416. 33328045 10.1016/S2589-7500(20)30159-X

[19] Bruce C , Prassas I , Mokhtar M *et al*. Transforming diagnostics: The implementation of digital pathology in clinical laboratories. Histopathology 2024; 85; 207–214. 38516992 10.1111/his.15178

[20] Eloy C , Vale J , Curado M *et al*. Digital Pathology workflow implementation at IPATIMUP. Diagnostics 2021; 11; 2111. 34829458 10.3390/diagnostics11112111 PMC8620597

[21] Stathonikos N , Nguyen TQ , Spoto CP , Verdaasdonk MAM , van Diest PJ . Being fully digital: Perspective of a Dutch academic pathology laboratory. Histopathology 2019; 75; 621–635. 31301690 10.1111/his.13953 PMC6856836

[22] Woods L , Eden R , Green D *et al*. Impact of digital health on the quadruple aims of healthcare: A correlational and longitudinal study (Digimat study). Int. J. Med. Inform. 2024; 189; 105528. 38935999 10.1016/j.ijmedinf.2024.105528

[23] Kearney V , Chan JW , Valdes G , Solberg TD , Yom SS . The application of artificial intelligence in the IMRT planning process for head and neck cancer. Oral Oncol. 2018; 87; 111–116. 30527225 10.1016/j.oraloncology.2018.10.026

[24] Brady AP , Neri E . Artificial intelligence in radiology—Ethical considerations. Diagnostics 2020; 10; 231. 32316503 10.3390/diagnostics10040231 PMC7235856

[25] Brady AP , Allen B , Chong J *et al*. Developing, purchasing, implementing and monitoring AI tools in radiology: Practical considerations. A multi‐society statement from the ACR, CAR, ESR, RANZCR & RSNA. J. Am. Coll. Radiol. 2024; 21; 1292–1310. 38276923 10.1016/j.jacr.2023.12.005

[26] Misra R , Keane PA , Hogg HDJ . How should we train clinicians for artificial intelligence in healthcare? Future Healthc J 2024; 11; 100162. 39371537 10.1016/j.fhj.2024.100162 PMC11452832

[27] Williams BJ , Treanor D . Practical guide to training and validation for primary diagnosis with digital pathology. J. Clin. Pathol. 2020; 73; 418–422. 31784420 10.1136/jclinpath-2019-206319

[28] Evans AJ , Brown RW , Bui MM *et al*. Validating whole slide imaging Systems for Diagnostic Purposes in Pathology: Guideline update from the College of American Pathologists in collaboration with the American Society for Clinical Pathology and the Association for Pathology Informatics. Arch. Pathol. Lab Med. 2021; 146; 440–450. 10.5858/arpa.2020-0723-CP 34003251

[29] Reis‐Filho JS , Kather JN . Overcoming the challenges to implementation of artificial intelligence in pathology. J. Natl. Cancer Inst. 2023; 115; 608–612. 36929936 10.1093/jnci/djad048 PMC10248832

[30] Jahn SW , Plass M , Moinfar F . Digital Pathology: Advantages, limitations and emerging perspectives. J. Clin. Med. 2020; 9; 3697. 33217963 10.3390/jcm9113697 PMC7698715

[31] The Royal College of Pathologists of Australasia . Guideline—Artificial Intelligence in Pathology, Version 1. Sydney: RCPA, 2024.

[32] Hanna MG , Olson NH , Zarella M *et al*. Recommendations for performance evaluation of machine learning in Pathology: A concept paper from the College of American Pathologists. Arch. Pathol. Lab Med. 2023; 148; e335. 10.5858/arpa.2023-0042-CP 38041522

[33] Marletta S , Eccher A , Martelli FM *et al*. Artificial intelligence–based algorithms for the diagnosis of prostate cancer: A systematic review. Am. J. Clin. Pathol. 2024; 161; 526–534. 38381582 10.1093/ajcp/aqad182

[34] Steiner DF , Nagpal K , Sayres R *et al*. Evaluation of the use of combined artificial intelligence and pathologist assessment to review and grade prostate biopsies. JAMA Netw. Open 2020; 3; e2023267. 33180129 10.1001/jamanetworkopen.2020.23267 PMC7662146

[35] Chatrian A , Colling RT , Browning L *et al*. Artificial intelligence for advance requesting of immunohistochemistry in diagnostically uncertain prostate biopsies. Mod. Pathol. 2021; 34; 1780–1794. 34017063 10.1038/s41379-021-00826-6 PMC8376647

[36] Satturwar S , Parwani AV . Artificial intelligence‐enabled prostate cancer diagnosis and prognosis: Current state and future implications. Adv. Anat. Pathol. 2024; 31; 136–144. 38179884 10.1097/PAP.0000000000000425

[37] van Dooijeweert C , Flach RN , ter Hoeve ND *et al*. Clinical implementation of artificial‐intelligence‐assisted detection of breast cancer metastases in sentinel lymph nodes: The CONFIDENT‐B single‐center, non‐randomized clinical trial. Nat. Can. 2024; 5; 1195–1205. 10.1038/s43018-024-00788-z PMC11358151 38937624

[38] Drogt J , Milota M , Vos S , Bredenoord A , Jongsma K . Integrating artificial intelligence in pathology: A qualitative interview study of users' experiences and expectations. Mod. Pathol. 2022; 35; 1540–1550. 35927490 10.1038/s41379-022-01123-6 PMC9596368

