# Supplementary material for: Practical implementation of AI in a non‐academic, non‐commercial Pathology laboratory: Real world experience and lessons learned
Source: Histopathology. 2025 Jun 17;87(5):635–46. doi: 10.1111/his.15481 (PMC12522029; doi:10.1111/his.15481)
Supplement: Supplementary file 1 — Data S1. [file HIS-87-635-s001.docx]

# Supplementary tables

| **Cloud** | **On premises** |
| --- | --- |
| More flexibility on scalability of computer power and storage capacity | Less flexibility on scalability of computer power and storage capacity |
| Sensitive patient data out of the physical lab | Greater control over sensitive patient data |
| Smoother update policy | Higher maintenance efforts |

*Supplementary table 1: high level differences between cloud and on premises installations*

| **Reducing costs** | **Patient experience** | **Provider experience** | **Population health** |
| --- | --- | --- | --- |
| Increased accuracy | Decrease turnaround time | Decreased working pressure by AI support | Increased accuracy |
| Decreased amount of extra techniques | Increased trust in diagnosis by AI support for health care provider | Administrative support by AI |  |
| Automation of workflow by AI enhanced workflows |  |  |  |

Supplementary table 2: Implementation of AI following the quadruple aim scheme

| **Content of the AI training** |
| --- |
| - General information about AI technology |
| - Technical instructions regarding the specific functionalities of the AI tool |
| - Type of data utilized to train the algorithm |
| - Formulation of probability cut-offs |
| - Evaluation metrics such as Area Under the Curve (AUC), Sensitivity and Specificity |

Supplementary table 3: content of the AI training

| **Amount of IHC per case** | **n** | **Mean (#)** | **p-value** |
| --- | --- | --- | --- |
| Q1 2023 | 170 | 2.98 ±  0.16 | 0.001 |
| Q1 2024 | 155 | 2.26 ± 0.18 ↓ |  |
| Q2 2023 | 112 | 3.09 ± 0.19 | <0.001 |
| Q2 2024 | 122 | 2.07 ± 0.19 ↓ |  |
| Q3 2023 | 127 | 3.16 ± 0.21 | <0.001 |
| Q3 2024 | 140 | 1.96 ± 0.19 ↓ |  |
| Q4 2023 | 119 | 2.43 ± 0.19 | 0.003 |
| Q4 2024 | 109 | 1.65 ± 0.20 ↓ |  |
| 2023 total | 528 | 2.92 ± 0.09 | <0.001 |
| 2024 total | 526 | 2.00 ± 0.09 ↓ |  |

Supplementary table 4: Mean amount of Immunohistochemical tests (IHC) per prostate biopsy case in the periods before (2023) and following (2024) the integration of an artificial intelligence system for prostate biopsy evaluation, presented quarterly and annually.

| **Turn Around Time** | **n** | **Mean (days)** | **p-value** |
| --- | --- | --- | --- |
| Q1 2023 | 170 | 1.65 ± 0.08 | 0.014 |
| Q1 2024 | 155 | 1.94 ± 0.10 ↑ |  |
| Q2 2023 | 112 | 2.13 ± 0.10 | 0.009 |
| Q2 2024 | 122 | 1.78 ± 0.11 ↓ |  |
| Q3 2023 | 127 | 2.18± 0.11 | <0.001 |
| Q3 2024 | 140 | 1.58 ± 0.09 ↓ |  |
| Q4 2023 | 119 | 1.96 ± 0.11 | 0.153 |
| Q4 2024 | 109 | 1.79 ± 0.13 ↓ |  |
| 2023 | 528 | 1.95 ± 0.05 | 0.009 |
| 2024 | 526 | 1.78 ± 0.05 ↓ |  |

Supplementary table 5: Mean Turn Around Time (TAT) per prostate biopsy case in the periods before (2023) and following (2024) the integration of an artificial intelligence system for prostate biopsy evaluation, presented quarterly and annually.
